# Morphological differences in myofibre size and shape: A comparative study of the soleus, gastrocnemius, triceps brachii and vastus lateralis in humans and mice

**DOI:** 10.1111/joa.70025

**Published:** 2025-07-17

**Authors:** Casper Soendenbroe, Rene B. Svensson, Bettina Mittendorfer, S. Peter Magnusson, Abigail L. Mackey, Jesper L. Andersen

**Affiliations:** ^1^ Department of Orthopedic Surgery, Institute of Sports Medicine Copenhagen Copenhagen University Hospital – Bispebjerg and Frederiksberg Copenhagen Denmark; ^2^ Department of Clinical Medicine, Center for Healthy Aging University of Copenhagen Copenhagen Denmark; ^3^ Department of Medicine and Nutrition, School of Medicine University of Missouri Columbia Missouri USA; ^4^ Department of Exercise Physiology, School of Medicine University of Missouri Columbia Missouri USA

**Keywords:** locomotion, myofibre morphology, shape factor, skeletal muscle

## Abstract

Certain skeletal muscles are specialized for their functional roles, yet direct comparisons of cellular morphology of distinct muscles beyond fibre type distribution are limited. This study investigated myofibre morphology in predominantly slow, fast and mixed fibre muscles in humans and mice, with the aim of establishing reference values for muscle‐specific myofibre size and shape. Nine healthy young men (Age: 26 ± 1 years, BMI: 23 ± 1 kg/m^2^) had muscle biopsies taken from soleus, triceps brachii and vastus lateralis muscles. Additionally, the soleus and gastrocnemius muscles were harvested from 7 male C57BL/6 mice. Muscle samples were analysed by ATPase (human) or immunofluorescence (mouse) stainings of fibre type specific cross‐sectional area, perimeter and Shape Factor Index (SFI; fibre perimeter^2^/4 × π × fibre cross‐sectional area). In humans, type I fibres had 30%–40% larger CSA and 4%–7% higher SFI in soleus (1.54 ± 0.06) compared to triceps brachii (1.47 ± 0.05) and vastus lateralis (1.43 ± 0.04). Type IIa fibres SFI were 10%–11% higher in soleus (1.61 ± 0.08) compared to triceps brachii (1.45 ± 0.04) and vastus lateralis (1.45 ± 0.08). Soleus type I fibres were more heterogeneous in terms of size and shape compared to other muscles. Analyses of mouse muscle showed a similar pattern, in that CSA and SFI were higher in type I and IIa fibres of the soleus compared to the gastrocnemius. These findings suggest a consistent morphological characteristic of soleus fibres across species, with potentially important implications for future biomedical research.

## Graphical abstract

Myofibre size and shape were investigated in functionally distinct muscles of humans and mice using ATPase histochemistry and immunofluorescence. In both species, soleus myofibres were found to be larger, more irregularly shaped, and more heterogeneous compared to other muscles. These findings may have implications for biomedical research and potential clinical relevance.

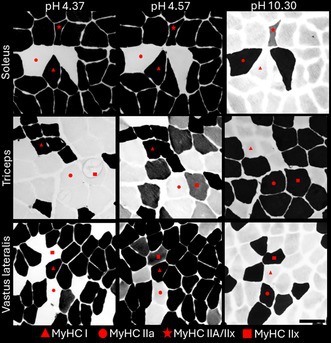

## 1 INTRODUCTION

In humans, the triceps brachii, soleus and vastus lateralis are three structurally, functionally and molecularly distinctive skeletal muscles each specialized for their respective tasks (Lieber & Fridén, 2000). The triceps brachii is a tri‐headed, fusiform, bi‐articular muscle composed predominately of fast‐twitch muscle fibres (type IIa, IIx). In contrast, the soleus is a multi‐pennate muscle composed predominately of slow‐twitch muscle fibres (type I). The vastus lateralis represents, in terms of fibre type composition, a mixture of approximately even proportions of slow and fast‐twitch muscle fibres, albeit with substantial interindividual variation (Saltin et al., 1977). Additionally, there is also specialization in fiber type distribution between species, which reflects evolutionary adaptations to different locomotor demands (Queeno et al., 2023). Certain muscles are uniquely specialized for their specific functions. The triceps brachii is seldom activated during daily life, but must generate moderate to high muscle activity in short bursts during specific activities, such as pushing or breaking a fall (Yamazaki et al., 1993). In contrast, the soleus is active during standing, walking and running and can sustain low to moderate muscle activity for prolonged periods of time (Gazendam & Hof, 2007; Winter & Yack, 1987). Accordingly, its overall architecture allows for large force generation combined with low excursion (change in fibre length per joint movement) (Ward et al., 2009). The vastus lateralis covers the whole spectrum of muscle activity, from mild activity during walking (Winter & Yack, 1987), to maximum activity during sprinting or jumping (Ebben et al., 2008).

Recent studies have shown that different skeletal muscles show discordant responses to disuse, exercise, growth and ageing. Disuse, either as bed rest or limb immobilization of healthy individuals, causes greater declines in the volume of the triceps surae muscles (soleus and gastrocnemius) compared to knee extensors and dorsiflexors (Hardy et al., 2022). Given that the soleus muscle constitutes close to two‐thirds of triceps surae muscle volume (Kolk et al., 2015), substantial atrophy of the triceps surae with disuse would likely also involve the soleus. Studies reporting muscle‐specific changes within the triceps surae with disuse are scarce, yet both Seynnes et al. (2008) and Belavý et al. (2009) observed a similar magnitude of atrophy for soleus and gastrocnemius in healthy individuals (Belavý et al., 2009; Seynnes et al., 2008). Additionally, atrophy of soleus myofibres has been reported after only 6 days of bedrest in young healthy individuals (Hendrickse et al., 2022). In the opposite scenario, with increased usage elicited through long‐term heavy resistance exercise, the triceps surae has been described as relatively unresponsive in terms of hypertrophy (Trappe et al., 2004). Accordingly, reported changes in muscle mass and muscle strength following long‐term resistance exercise interventions are mixed, with some studies showing improvements in the triceps surae that are of comparable magnitude to the knee extensors and others showing a dampened response (Ferri et al., 2003). Muscle specification during growth has also been reported (Siebert et al., 2017). Finally, in ageing, there is substantial evidence in both humans (Naruse et al., 2023, p. 202) and rats (Burke et al., 2021) that the soleus is relatively spared from age‐related atrophy compared to other lower limb muscles, likely owing to its type I myofibre dominance. Taken together, the underlying reasons for these discordant changes with disuse, exercise and ageing are not entirely clear, but differences in muscle fibre composition and morphology are plausible factors.

We have recently shown, using a cohort of 197 men and women covering an age span of 20–97 years, that myofibre shape was linked to measures of muscle strength and mass (Soendenbroe et al., 2024). Myofibre shape was expressed as the Shape Factor Index (SFI; fibre perimeter^2^/4 × π × fibre cross‐sectional area), which is a dimensionless expression of shape that, using the cross‐sectional area and perimeter of a myofibre, can be used to indicate the degree of deviation from normal fibre morphology. We observed significant differences in SFI between type I and II myofibres in young healthy individuals (1.34 ± 0.04 and 1.40 ± 0.04 [mean ± SD], respectively). Crucially, only vastus lateralis muscle biopsies were investigated, meaning that potential inter‐muscle specialization in myofibre shape remains unexplored. Importantly, muscle biopsies are used for diagnosis of neuromuscular diseases when less invasive approaches are insufficient or inconclusive (Ross et al., 2023), and in certain cases, this may necessitate sampling of the affected muscles in the arms or legs (Joyce et al., 2012). Such muscles include the triceps brachii and the soleus, which can be biopsied both effectively and safely (Cotter et al., 2013; Deschrevel et al., 2024). In rodent models, the soleus is often chosen as a representative slow‐twitch muscle due to its predominance of type I fibres. As such, specific SFI values for these highly specialized muscles are of scientific and clinical value.

The aim of this study was to, in both humans and mice, investigate myofibre morphology in slow (soleus for both species), fast (triceps brachii for humans and gastrocnemius for mice) and mixed (vastus lateralis for humans) muscles, with the goal of establishing SFI reference values for these distinct muscle types. We hypothesized that myofibres in predominantly fast‐twitch muscles would exhibit higher SFI values compared to those in slow‐twitch muscles.

## 2 METHODS

### 2.1 Human participants

The study was approved by local ethics committees (Copenhagen and Frederiksberg Communities, Denmark) and was conducted in accordance with the Declaration of Helsinki. Written informed consent was obtained from all participants. Nine recreationally active, healthy, young (Age: 26 ± 1 years), normal weight (BMI: 23 ± 1 kg/m^2^) men were enrolled in the project. All participants underwent a medical examination prior to enrollment. Muscle‐specific protein synthesis rates have been previously reported for these individuals (Mittendorfer et al., 2005).

### 2.2 Study design

Participants were instructed to follow their regular diet and to avoid hard exercise for the 3 days leading up to the study. On the day of the study, participants arrived fasted at 07.00 h. At 08.00 h, a primed 6‐h‐long constant infusion of stable isotope labelled tracer was initiated. At 11.00 h, a 3‐h‐long infusion of a mixed balanced amino acid solution was initiated. Muscle biopsies were obtained under local anaesthesia (2% Lidocaine), using the Bergström biopsy technique (Bergstrom, 1975), from the soleus, triceps brachii and vastus lateralis (mid‐thigh) in randomized order at both 11.00 h and 14.00 h (6 biopsies in total). Vastus lateralis biopsies were taken from the mid‐thigh region, and triceps brachii biopsies were taken from the mid‐region. Soleus biopsies were taken by entering distally and laterally to the gastrocnemius muscle, as described by others (Cotter et al., 2013). Repeated biopsies were taken through different incision sites and combined for histological analysis. Extracted tissue was carefully aligned, embedded in Tissue Tek (Sakura Finetek, Zoeterwoude, the Netherlands) and frozen in isopentane (JT Baker) pre‐cooled in liquid nitrogen. Samples were stored at −80°C.

### 2.3 Animals

Seven male C57BL/6 mice (Janvier), that were part of a larger study investigating the effect of ageing and different modes of exercise training in muscle morphology (Olesen et al., 2021), were used in the present study. All experiments were conducted in accordance with Danish legislation (Amendment #1306 of November 23, 2007), and the project was approved by the Danish Animal Inspectorate, Ministry of Justice (#2014‐15‐020100326). Animals were housed individually in a 12 h light–dark cycle, with access to tap water and standard chow, but without access to a running wheel. Following euthanization by cervical dislocation, the left soleus and gastrocnemius muscles were dissected, embedded in Tissue Tek and frozen in isopentane (JT Baker) pre‐cooled in liquid nitrogen. Samples were stored at −80°C.

### 2.4 ATPase and immunofluorescence stainings

Frozen human and mouse muscle samples were sectioned at 10 μm thickness using a cryostat at −20°C. Human samples were subjected to ATPase histochemistry at pH 9.40 after pre‐incubation at pH 4.37, 4.60 and 10.30. In brief, three serial sections placed on individual glass slides are incubated at the three different pH values were then transferred to pH 9.40 and subsequently visualized using microscopy For more details on the differentiation of the various fiber types see Andersen & Aagaard (2000). Mouse samples, collected at the middle of the soleus and gastrocnemius muscles, were subjected to immunofluorescent staining on two subsequent sections with Laminin (RRID:AB_2313665, Z0097, Dako) and either MyHC I (RRID:AB_2235587, BA.D5, DSHB) and MyHC IIb (RRID:AB_2811120, BF‐F3, DSHB) or MyHC IIa (RRID:AB_2147165, SC‐71, DSHB) and MyHC IIx (RRID:AB_1157897, 6H1, DSHB). Samples were pre‐blocked with 2.5% goat serum albumin in tris‐buffered saline for 30 min, covered with mounting medium (ProLong Gold Anti‐fade, Invitrogen A/S, Taastrup, Denmark) and coverslips, dried in the dark and stored at −80°C.

### 2.5 Microscopy and image analyses

**FIGURE 1:**
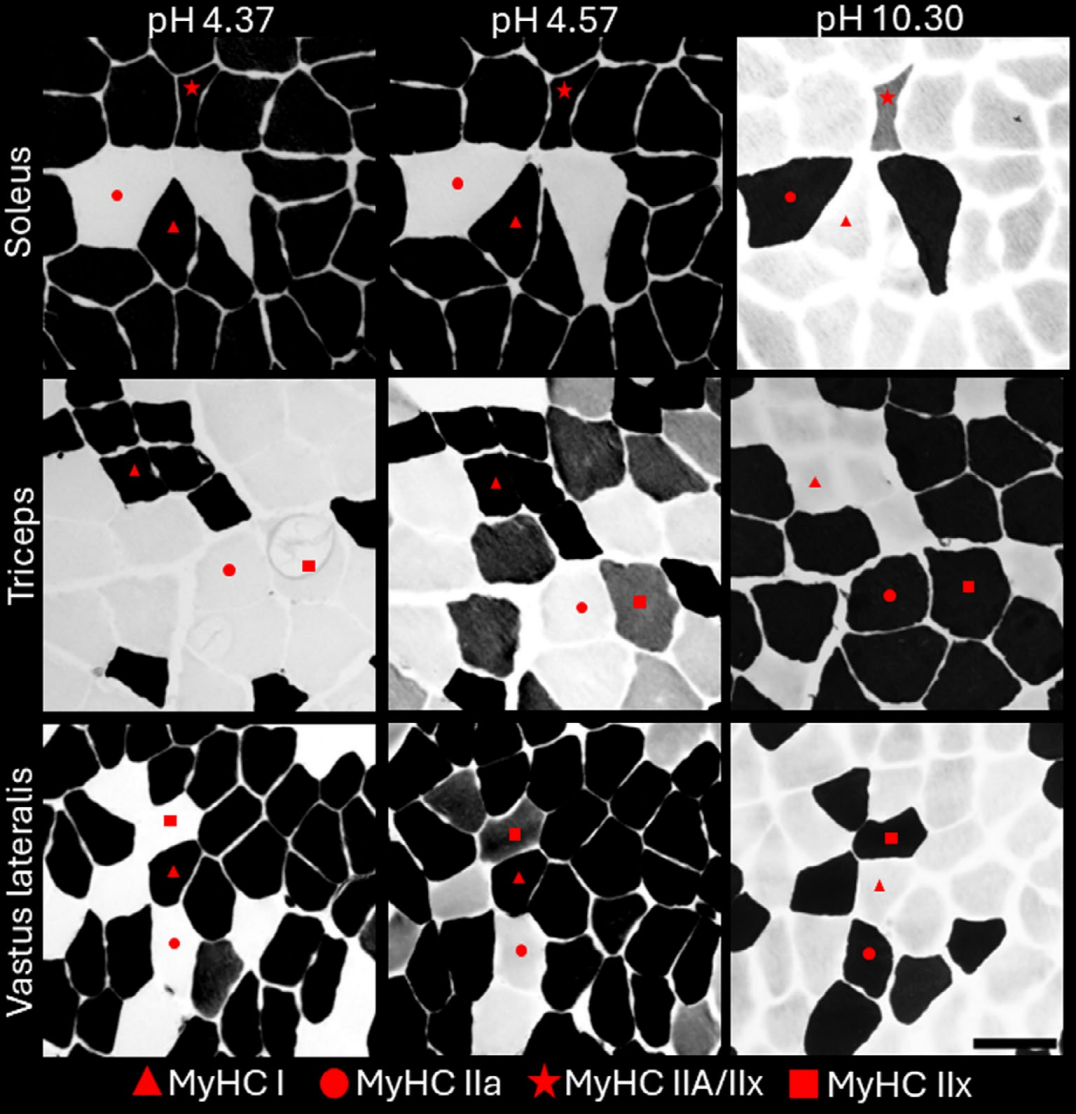
Serial sections of soleus, triceps brachii and vastus lateralis muscle biopsies stained with ATPase at 4.37 pH (Dark: Type I), 4.57 pH (Dark: Type I and IIx) and 10.30 pH (Dark: Type IIa and IIx). Red symbols are used to indicate different fibre types. In soleus 10.30 a type I/IIa hybrid can be seen. Scale bar equals 100 μm.

ATPase‐stained human muscle samples were analysed by manually outlining myofibre membrane (TEMA, Scan Beam ApS, Hadsund, Denmark), and subsequently classifying them as MyHC type I, I/IIa, IIa, IIa/IIx and IIx (Figure 1). The two biopsies (taken at 11.00 h and 14.00 h) from each muscle showed good agreement in terms of fibre type distribution and were therefore analysed as one in the present study. The fibre type percentages and area percentages have previously been reported (Mittendorfer et al., 2005), but the former is reported in Table 1 to aid in interpretation. Type I/IIa fibres were only observed in one participant, meaning that this category could not be reliably used in the subsequent analyses and was therefore excluded.

**TABLE 1:** Number of myofibres counted and fibre type percentages of each type for each muscle. Data are means ± SD with minimum and maximum.

				Type I		Type IIa		Type IIa/IIx		Type IIx		Type IIx/IIb		Type IIb	
				Mean ± SD	Min – Max	Mean ± SD	Min – Max	Mean ± SD	Min – Max	Mean ± SD	Min – Max	Mean ± SD	Min – Max	Mean ± SD	Min – Max
Human	Soleus	180 ± 73 [89–300]	*n*	153 ± 75	60–274	20 ± 16	0–41	3 ± 2	0–6	4 ± 3	0–9	N/A		N/A	
			%	82 ± 14	55–98	13 ± 11	0–34	2 ± 2	0–6	3 ± 3	0–10				
	Triceps brachii	212 ± 81 [83–321]	*n*%	46 ± 17	13–65	71 ± 32	19–123	19 ± 10	2–32	75 ± 52	1–159	N/A		N/A	
			%	22 ± 7	16–34	34 ± 14	21–69	9 ± 4	2–15	34 ± 19	1–59				
	Vastus lateralis	156 ± 61 [73–260]	*n*	92 ± 38	42–152	36 ± 26	8–97	8 ± 5	0–16	18 ± 12	3–35	N/A		N/A	
			%	59 ± 10	39–71	21 ± 9	11–37	6 ± 4	0–14	13 ± 9	2–28				
Mice	Soleus	652 ± 187 [383–915]	*n*	262 ± 92	177–441	363 ± 108	192–485	45 ± 38	12–113	24 ± 19	1–50	1 ± 1	0–2	2 ± 6	0–15
			%	40 ± 6	33–48	56 ± 5	49–61	N/A		4 ± 3	0–8	N/A		0 ± 1	0–3
	Gastrocnemius	1126 ± 443 [365–1716]	*n*	29 ± 22	2–59	211 ± 93	77–326	34 ± 19	9–59	125 ± 51	54 188	17 ± 13	6–40	761 ± 305	205–1150
			%	2 ± 1	0–4	19 ± 5	10–26	N/A		11 ± 2	9–15	N/A		67 ± 8	56–80

Immunofluorescence‐stained mouse muscle samples were imaged, using a 0.50 NA/20× objective, BX51 microscope and DP71 camera (Olympus), sequentially and with ~10% overlap. Sequential images were stitched into one seamless image and analysed using a custom‐made semi‐automated macro in ImageJ. Fibres were classified as type I, I/IIa, IIa, IIa/IIx, IIx, IIx/IIb or IIb. The fibre type percentages and cross‐sectional areas have previously been reported (Olesen et al., 2021), but the former is reported again to aid in interpretation. Type I/IIa were present in low numbers (1–17) in 8 out of 14 muscles and are not analysed further.

**FIGURE 2:**
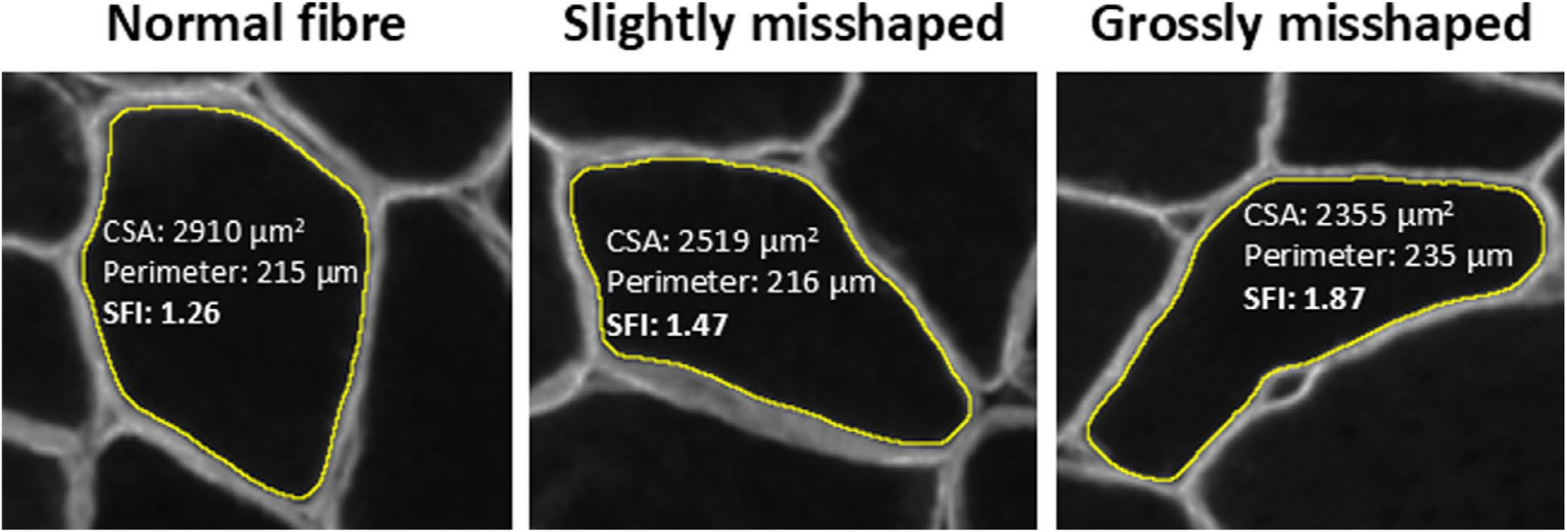
CSA, perimeter and SFI values for myofibres with normal, slightly misshaped and grossly misshaped morphology. CSA, cross‐section area; SFI, Shape Factor Index.

For both human and mice samples, fibre type‐specific cross‐sectional area (CSA) and perimeter were analysed. Deviations in myofibre shape were analysed using the Shape Factor Index (SFI; fibre perimeter^2^/4 × π × fibre cross‐sectional area), which, independent of fibre size, indicates the degree of deformity of a fibre. A minimum of 5 fibres of a given type was required from each sample to be included in summary data (means and deviations). The exact number of biological replicates for each muscle is provided in respective figure legends. Fibre heterogeneity in size and shape was explored in two ways. Firstly, by evaluating the distribution of SFI and CSA, in 0.10 increments from <1.0 to >2.0 and 1000 μm^2^ increments from 0 to >15,000 μm^2^, respectively. Secondly, by calculating the coefficient of variation (CV) within each sample for SFI and CSA (Roberts et al., 2018). Representative examples of myofibres with normal (SFI 1.20–1.45), slightly misshaped (1.46–1.70) or grossly misshaped (>1.71) morphology are provided in Figure 2.

### 2.6 Statistics

Data are shown as means with individual, connected values or means with standard deviations. Data from human muscle were, when three muscles were represented, analysed using mixed‐effects models with muscle as the independent variable and the Tukey post hoc test. When only two muscles were represented, two‐tailed paired *t*‐tests were conducted. Data from mouse muscle were analysed using two‐tailed paired *t*‐tests. Graphs and statistical analyses were conducted in Prism (v.10, GraphPad Software). Statistical significance was set at 0.05, and tendencies are given.

## 3 RESULTS

### 3.1 Soleus myofibres possess unique traits in humans

**FIGURE 3:**
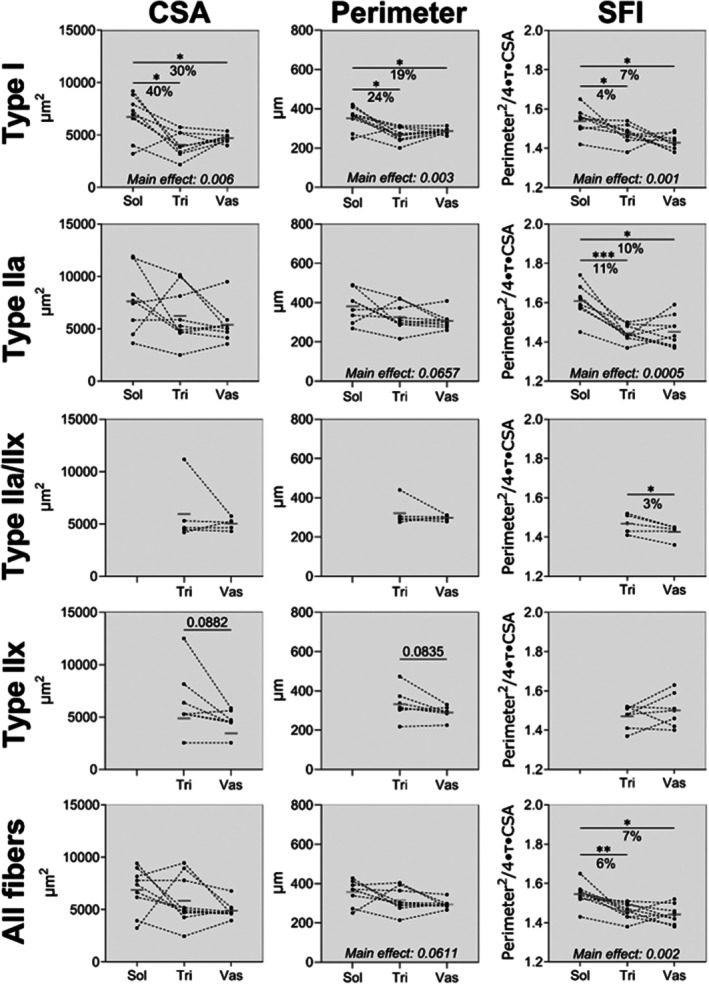
CSA, perimeter and SFI of each fibre type in soleus, triceps brachii and vastus lateralis of 9 young healthy men. Data are shown as connected individual values and group means (grey line). Data were analysed, when three muscles were represented, by mixed‐model repeated measures ANOVA (muscle as factor), using Tukey's post hoc, and when two muscles were represented by paired two‐tailed *t*‐tests. N: Type I = 9 in all muscles. Type IIa = 8 in Sol, 9 in Tri and Vas. Type IIa/IIx = 5 in Tri and Vas. Type IIx = 7 in Tri and Vas. CSA, cross‐section area; SFI, shape factor index; Sol, soleus; Tri, triceps brachii; Vas, vastus lateralis.

Type IIa/IIx and IIx fibres were scarce in soleus and are therefore not reported. As shown in Figure 3, an effect of muscle was observed for type I CSA (main effect of muscle, *F* (1.39, 11.09) = 10.07, *p* = 0.006), with post hoc test revealing type I fibres in soleus to be 40% (*p* < 0.05) and 30% (*p* < 0.05) larger on average than type I fibres in triceps brachii and vastus lateralis, respectively. Similarly, perimeter was also greater in soleus compared to other muscles (main effect of muscle, *F* (1.48, 11.86) = 11.44, *p* = 0.003), but to a lesser degree. As a result (*F* (1.74, 20.91) = 10.47, *p* = 0.001), SFI was found to be 4% (*p* < 0.05) and 7% (*p* < 0.05) higher in type I in soleus compared to triceps brachii and vastus lateralis, respectively. Type IIa SFI (*F* (1.55, 11.66) = 17.98, *p* = 0.0005) was 11% (*p* < 0.05) and 10% (*p* < 0.05) higher in soleus compared to triceps brachii and vastus lateralis, respectively. The updated SFI values for soleus in young, healthy muscle are 1.54 ± 0.06 in type I and 1.61 ± 0.08 in type IIa fibres, as compared to 1.43 ± 0.04 in type I and 1.45 ± 0.08 in type IIa fibres in vastus lateralis. A 3% higher SFI was observed for IIa/IIx fibres in triceps brachii compared to vastus lateralis (*t*(4) = 3.38, *p* < 0.05). Across all fibres, a main effect of muscle was found (*F* (1.59, 12.70) = 12.02, *p* = 0.002), with soleus myofibre SFI values 6% (*p* < 0.005) and 7% (*p* < 0.05) higher than triceps brachii and vastus lateralis, respectively.

### 3.2 Myofibre heterogeneity

**FIGURE 4:**
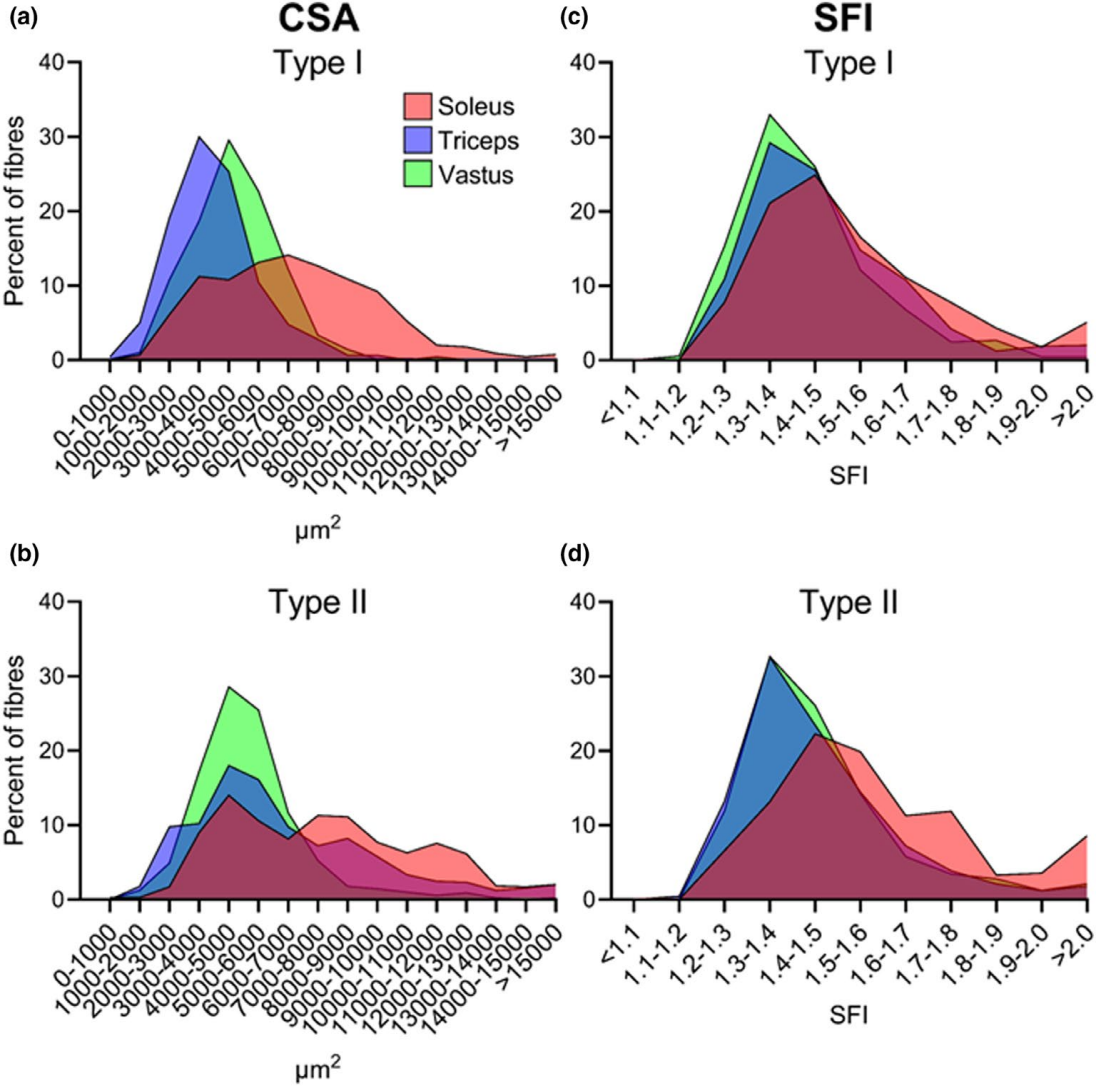
CSA and SFI distribution of type I and II fibres of soleus (red), triceps brachii (blue) and vastus lateralis (green) of 9 young healthy men. (a, b) Percentage of type I (a) and II (b) myofibres in 1000 μm^2^ increments of CSA. (c, d) Percentage of type I (c) and II (d) myofibres in 0.1 increments of SFI. Data are averages of all participants for each muscle and presented as means (see Figure S1 for version with error bars). N: Type I = 9 in all muscles. Type II = 7 in Sol, 9 in Tri and Vas. CSA, cross‐section area; SFI, Shape Factor Index.

**FIGURE 5:**
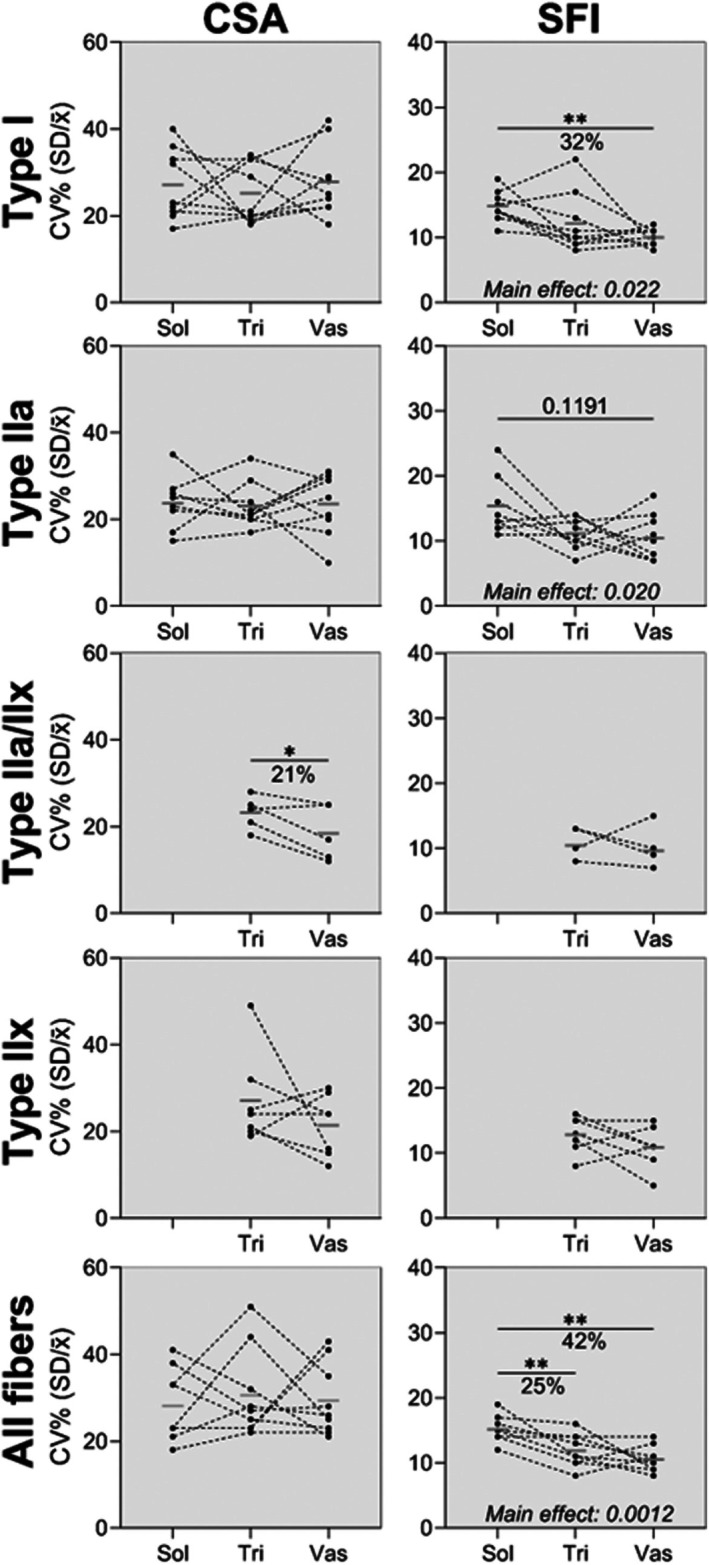
Coefficient of variation for CSA and SFI of each fibre type in the soleus, triceps brachii and vastus lateralis of 9 young healthy men. Data are shown as connected individual values and group means (grey line). Data were analysed, when three muscles were represented, by mixed‐model repeated measures ANOVA (muscle as factor), using Tukey's post hoc, and when two muscles were represented by paired two‐tailed *t*‐tests. N: Type I = 9 in all muscles. Type IIa = 8 in Sol, 9 in Tri and Vas. Type IIa/IIx = 5 in Tri and Vas. Type IIx = 7 in Tri and Vas. CSA, cross‐section area; SFI, Shape Factor Index; Sol, soleus; Tri, triceps brachii; Vas, vastus lateralis.

To explore fibre heterogeneity, histograms for CSA (1000 μm^2^ increments) and SFI (0.1 increments) were prepared. To maintain adequate fibre numbers, type IIa, IIa/IIx and IIx fibres were pooled into a collective type II category. As shown in Figure 4 and Figure S1, type I and II fibres in triceps brachii and vastus lateralis are relatively similar in terms of size and shape. In contrast, the soleus is rightward shifted, indicating larger CSA and higher SFI, and presents with flatter curves, indicating increased variability among fibres within each sample. To further investigate variability in size and shape, the CV was calculated for CSA and SFI. As shown in Figure 5, an effect of muscle was observed for SFI in both type I (*F* (1.60, 12.77) = 5.67, *p* = 0.022) and IIa (*F* (1.96, 22.49) = 4.74, *p* = 0.020) fibres, with post hoc testing showing 32% (*p* < 0.005) higher degree of variability in soleus type I SFI compared to vastus lateralis. The greater variability can be observed in Figure 1, where fibres in soleus vary considerably more in size and shape compared to fibres in triceps brachii and vastus lateralis.

### 3.3 Muscle specificity in myofibre size and shape in mice

**FIGURE 6:**
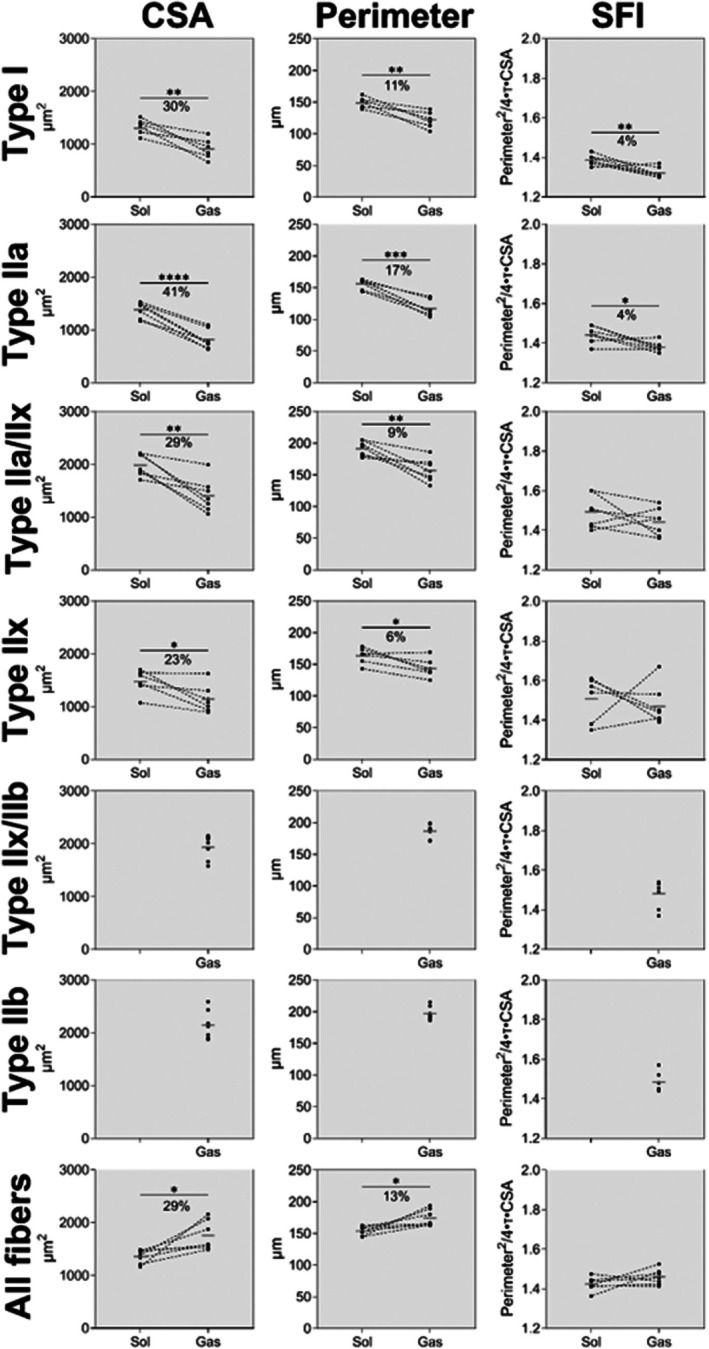
CSA, perimeter and SFI of each fibre type in soleus and gastrocnemius of 7 male 11‐month‐old C57BL/6 mice. Data are shown as connected individual values and group means (grey line). Data were analysed by paired two‐tailed *t*‐tests. *N* = 7. CSA, cross‐section area; SFI, Shape Factor Index; Sol, soleus; Gas, gastrocnemius.

**FIGURE 7:**
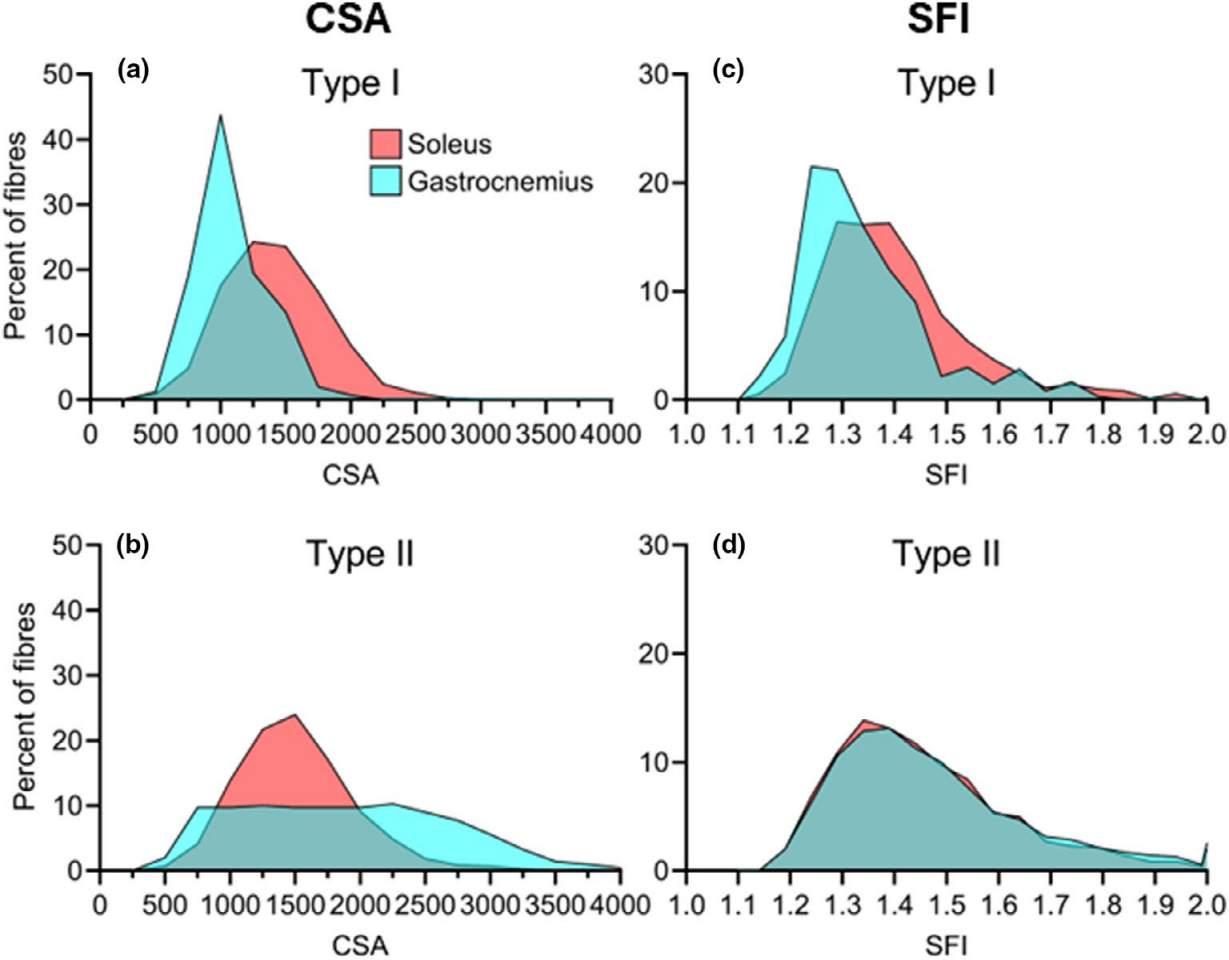
CSA and SFI distribution of type I and II fibres of soleus (red) and gastrocnemius (turquoise) of 7 male 11‐month‐old C57BL/6 mice. (a, b) Percentage of type I (a) and II (b) myofibres in 250 μm^2^ increments of CSA. (c, d) Percentage of type I (c) and II (d) myofibres in 0.1 increments of SFI. Data are averages of all mice for each muscle and presented as means. *N* = 7. CSA, cross‐section area; SFI, Shape Factor Index.

**FIGURE 8:**
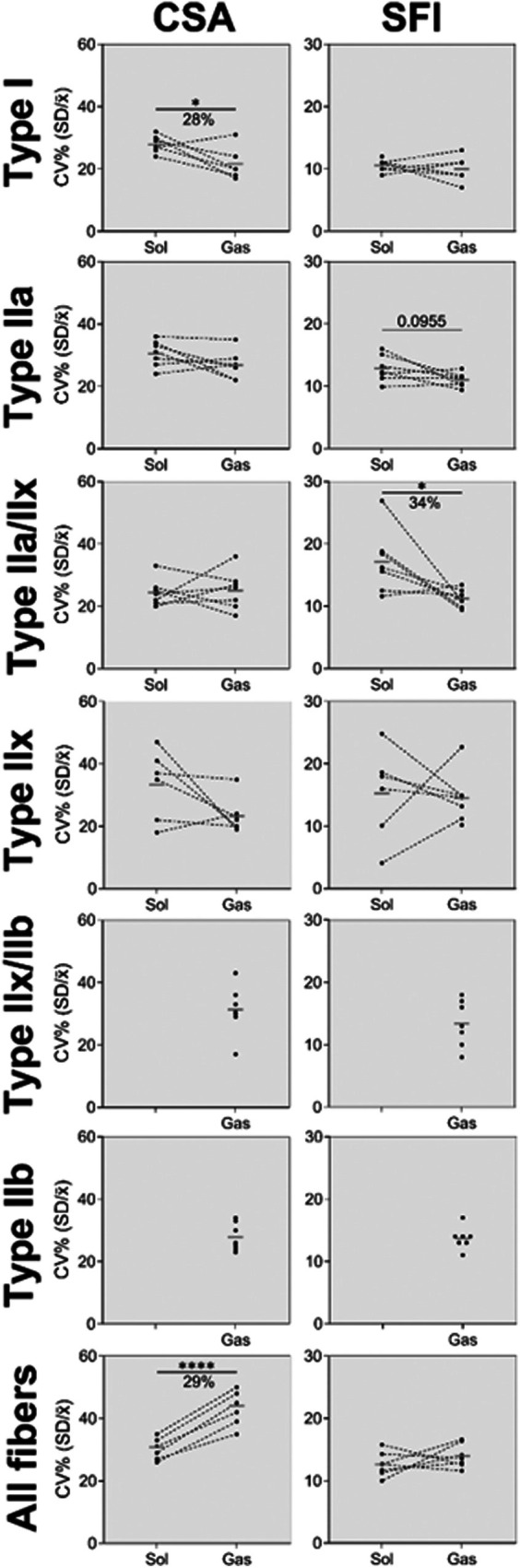
Coefficient of variation for CSA and SFI of each fibre type in the soleus (red) and gastrocnemius (turquoise) of 7 male 11‐month‐old C57BL/6 mice. Data are shown as connected individual values and group means (grey line). Data were analysed by paired two‐tailed *t*‐tests. *N* = 7. CSA, cross‐section area; SFI, Shape Factor Index; Sol, soleus; Gas, gastrocnemius.

Next, we wanted to examine if soleus myofibres in mice also showed unique traits. The number of fibres analysed is given in Table 1. Notably, there are substantial differences in fibre type distribution between soleus and gastrocnemius, but type IIa fibres are well‐represented in both muscles and likely serve as the best comparison. As shown in Figure 6, the CSA and perimeter were both greater in soleus compared to gastrocnemius, for type I (CSA, *t*(5) = 4.84, *p* < 0.005. Perimeter, *t*(5) = 4.77, *p* < 0.005), IIa (CSA, *t*(6) = 9.44, *p* < 0.0001. Perimeter, *t*(6) = 8.91, *p* < 0.0001), IIa/IIx (CSA, *t*(6) = 4.57, *p* = 0.004. Perimeter, *t*(6) = 4.31, *p* = 0.005) and IIx fibres (CSA, *t*(5) = 2.97, *p* = 0.031. Perimeter, *t*(5) = 3.17, *p* = 0.025). SFI was found to be greater in soleus compared to gastrocnemius for type I (*t*(6) = 3.99, *p* = 0.007) and IIa (*t*(6) = 3.11, *p* = 0.021) fibres. When combining all fibres, CSA and perimeter were found to be greater in gastrocnemius compared to soleus (CSA, *t*(6) = 3.15, *p* = 0.020. Perimeter, *t*(6) = 3.20, *p* = 0.019). This can be attributed to 67% ± 8% of fibres in gastrocnemius being type IIb, which are not present in soleus. As shown in Figures 7 and 8, the fibre type specialization is also apparent when exploring fibre heterogeneity. Larger CV is observed in soleus compared to gastrocnemius for some fibre types (type I CSA, *t*(5) = 2.61, *p* = 0.047. Type IIa/IIx SFI, *t*(6) = 2.59, *p* = 0.042). When evaluating all fibres combined, including type IIb fibres in gastrocnemius, CV for SFI is similar between muscles (*t*(6) = 1.08, *p* = 0.3203) and for CSA 29% (*t*(6) = 12.25, *p* < 0.001) larger in gastrocnemius compared to soleus (Figure 8). Looking specifically at type IIa myofibres, which is the fibre type that is most well‐represented in both muscles, a tendency was observed for SFI CV to be larger in soleus compared to gastrocnemius (*t*(6) = 1.98, *p* = 0.096), while no difference was observed for CSA (*t*(6) = 1.72, *p* = 0.136).

## 4 DISCUSSION

The gross morphology of certain skeletal muscles is specialized to facilitate the efficient execution of their primary functions. At the cellular level, this specialization is observed as the polarization of fibre type distribution (Burkholder et al., 1994). Across various mammalian species, the soleus and triceps brachii muscles occupy opposite ends of the fibre type spectrum (Queeno et al., 2023). Still, there is a scarcity of studies comparing functionally distinct muscles at the myofibre level beyond merely fibre type distribution. In this study, we provide evidence in humans that the size and shape of myofibres vary between functionally distinctive muscles. Specifically, soleus myofibres are larger, more deformed and more heterogeneous in shape and size compared to those of the triceps brachii and vastus lateralis. We extended this investigation by analysing two muscles with highly polarized fibre type distributions — the soleus and gastrocnemius — in mice. Again, we observed that slow‐twitch fibres were larger and more deformed in the soleus compared to gastrocnemius. Together, these findings reveal novel aspects of myofibre specialization with potential implications for future biomedical research, especially concerning muscle‐specific adaptations.

In human biomedical research, the vastus lateralis is the most frequently used muscle for biopsies, likely due to its superficial position, large size and relatively equal proportion of type I and II fibres, making it both accessible and safe for biopsy. The vastus lateralis also plays a key role in ambulation and its in vivo properties, such as strength and size, can be reliably evaluated (Soendenbroe, Dahl, et al., 2022a). The soleus muscle is mostly used for biopsies in studies investigating metabolic control (Hamilton et al., 2022), intramuscular signalling (Jensen et al., 2012), muscle injuries in the triceps surae (Häggmark & Eriksson, 1979) or protein turnover (Trappe et al., 2008), and how these processes function in a muscle dominated by slow‐twitch fibres. The soleus is also one of several muscles that may be biopsied when distal myopathy is suspected (Cotta et al., 2021). Common to all these research areas is their reliance on a thorough understanding of the muscle's gross and cellular morphology. The data presented here provide a foundational understanding of three distinct muscles and will hopefully aid future research in interpreting the outcomes of various interventions. A number of studies have quantitatively evaluated myofibre morphology in different human muscles; yet, as far as the authors are aware, only five studies have specifically included the soleus in these comparisons (Andersen & Kroese, 1978; Edström & Nyström, 1969; Gollnick et al., 1974; Luden et al., 2008; Sjøgaard, 1982). Briefly summarized, the soleus is dominated by type I fibres, and fibres of the soleus are found to be larger than fibres of other muscles. However, no information is available on fibre shape or heterogeneity in size and shape. Our results indicate that type I fibres in the soleus are 30%–40% larger than those in other muscles, in both humans and mice, highlighting a consistent morphological characteristic of the soleus across species. The level of deformity, assessed from the SFI, is 6%–7% higher in fibres of human soleus compared to triceps brachii and vastus lateralis.

These observations raise a key question: What functional or structural factors might account for these differences? Our previous work has demonstrated that ageing is associated with an increase in SFI of type II and, to a lesser degree, type I fibres (Soendenbroe et al., 2024). Specifically, the SFI of type II fibres in individuals above the age of 80 was 1.59 ± 0.11, as opposed to 1.40 ± 0.04 in young individuals (13.6% difference). To put this into context, the SFI of soleus type I fibres in the present study was 1.54 ± 0.06, meaning that they are close to the level observed in aged individuals, among which many were sarcopenic and/or geriatric patients. We speculated that the age‐related differences were driven by an ongoing process of denervation and reinnervation (Soendenbroe et al., 2019), whereby denervated fibres would undergo degradation and thereby lose key structural features, among them fibre shape (Viguie et al., 1997). Given that all enrolled participants in the present study were recreationally active and healthy, it appears unlikely that denervation is the main explanation for the high SFI in soleus muscle. An alternative explanation for the high SFI values in soleus compared to other muscles relates to its unique muscle architecture. Ward et al. (2009) examined the muscle architecture of 27 different lower extremity muscles obtained from 21 individuals (Ward et al., 2009). In comparison with 10 other muscles acting on the ankle joint, soleus was the largest and longest muscle, but also had the shortest sarcomere length and highest pennation angle (more than two‐fold higher than gastrocnemius). A high pennation angle enables denser packing of myofibres within a given volume, enhancing force production efficiency despite the shorter sarcomere length. This architecture is advantageous for the soleus's primary role as a postural muscle, enabling it to endure prolonged contractions with minimal fatigue. Finally, it should also be mentioned that long‐term heavy resistance exercise, which leads to increased muscle mass and muscle strength (Soendenbroe, Heisterberg, et al., 2022), causes SFI values of vastus lateralis myofibres to decline. This suggests that an increased demand for force production is met by an optimization of fibre shape. Yet, the soleus muscle, which is more‐or‐less constantly active throughout a day (Gazendam & Hof, 2007), presents with much higher SFI values than vastus lateralis. This pattern may suggest a dose–response relationship, where a moderate level of muscle activity supports optimal fibre morphology, while constant activation could impose a strain, contributing to the observed differences in SFI. Alternatively, it could mean that heavy resistance exercise, where a maximal load is imposed on the muscles, requires full motor unit activation, which is very different from the everyday activities experienced by soleus, where motor unit recruitment is more variable and task dependent. Given that training was not performed in the present study, it remains unknown whether the shape of fibres in soleus would normalize in a similar manner to what is observed in the vastus lateralis.

Another unique aspect of soleus morphology is that its myofibres are more variable in terms of size and shape. While the origin of this heterogeneity is not clear, abnormally large fibres have previously been observed amidst normal size fibres in the trapezius muscle of individuals suffering from myalgia (Andersen et al., 2008). This was suggested to reflect an adaptation to specific work demands, whereby few fibres had become overloaded. Whether the same applies to the soleus, in that few fibres are more frequently activated, is unclear, yet the trapezius myalgia situation was associated with chronic pain, which is not the case for the soleus. An alternative explanation relates to the process of embryogenesis. The first myofibres are formed during around week 7–9 in humans (Fidziańska, 1980). These fibres, termed primary generation, are followed a couple of weeks later by a second wave of fibres termed secondary generation (Condon et al., 1990). Secondary generation fibres form around the existing primary generation fibres, meaning that fibres of the two generations are interspersed among each other and not localized to specific regions of the muscle. A unique aspect of primary generation fibres is that they are committed to a given phenotype, whereas secondary generation are amendable to change of fibre type upon various cues (Blaauw et al., 2013). Secondary generation fibres can also more readily switch their myosin configuration during postnatal development (Schiaffino & Reggiani, 1996). Consequently, fibres determined by ATPase stainings to have the same phenotype actually constitute a diverse group of fibres with divergent pasts. It is tempting to hypothesize that the type I myofibres in the soleus with the highest CSA and SFI values might be secondary generation fibres that were originally type II fibres. How this phenotypic heterogeneity interacts with the structural consistency of lower‐ (Willingham et al., 2020) and higher‐order (Wick et al., 2018) hierarchical units remains an open question.

The present study is limited by relatively small sample sizes, both in relation to human (*n* = 9) and mice (*n* = 7) experiments, and only males were studied. Furthermore, given that the aim of the study was to investigate myofibre morphology in muscles with a slow, fast and mixed fibre type composition, this naturally means that it can be difficult to include enough type II fibres in a slow muscle for instance, whereby some types of fibres are represented by >100 individual myofibres, whereas others by only 10–20.

In conclusion, our findings from human muscle biopsies and mice muscles reveal that soleus myofibres exhibit unique morphological traits. Specifically, soleus myofibres were found to be larger, more deformed and more heterogeneous compared to those in the gastrocnemius, triceps brachii and vastus lateralis. These distinctive characteristics suggest that the soleus is an atypical muscle, raising questions about its suitability as the sole muscle of choice in biomedical research. Future research should investigate a broader range of muscles and species to determine whether these morphological distinctions are conserved across varying functional demands and evolutionary lineages.

## AUTHOR CONTRIBUTIONS

All authors conceived and designed research; CS, RBS, BM and JLA performed experiments; CS, RBS and JLA analysed data; CS and JLA interpreted results of experiments; CS prepared figures; CS drafted manuscript; CS and JLA edited and revised manuscript; All authors approved the final version of the manuscript.

## CONFLICT OF INTEREST STATEMENT

No conflicts of interest, financial or otherwise, are declared by the authors.

## Supporting information


**Figure S1.** CSA and SFI distribution of type I and II fibres of soleus (red), triceps brachii (blue) and vastus lateralis (green) of 9 young healthy men. (A‐B) Percentage of type I (A) and II (B) myofibres in 1000 μm^2^ increments of CSA. (C‐D) Percentage of type I (C) and II (D) myofibres in 0.1 increments of SFI. Data are averages of all participants for each muscle and presented as means ± SEM. N: Type I = 9 in all muscles. Type II = 7 in Sol, 9 in Tri and Vas. Abbreviations: CSA, cross‐section area. SFI, shape factor index.

## Data Availability

The data that support the findings of this study are available from the corresponding author upon reasonable request.

## References

[joa70025-bib-0001] Andersen, J.L. & Aagaard, P. (2000) Myosin heavy chain IIX overshoot in human skeletal muscle. Muscle & Nerve, 23, 1095–1104. Available from: 10.1002/1097-4598(200007)23:7<1095::AID-MUS13>3.0.CO;2-O 10883005

[joa70025-bib-0002] Andersen, L.L. , Suetta, C. , Andersen, J.L. , Kjær, M. & Sjøgaard, G. (2008) Increased proportion of megafibers in chronically painful muscles. Pain, 139, 588–593. Available from: 10.1016/j.pain.2008.06.013 18701218

[joa70025-bib-0003] Andersen, P. & Kroese, A.J. (1978) Capillary supply in soleus and gastrocnemius muscles of man. Pflugers Archiv: European Journal of Physiology, 375, 245–249. Available from: 10.1007/BF00582437 567793

[joa70025-bib-0004] Belavý, D.L. , Miokovic, T. , Armbrecht, G. , Richardson, C.A. , Rittweger, J. & Felsenberg, D. (2009) Differential atrophy of the lower‐limb musculature during prolonged bed‐rest. European Journal of Applied Physiology, 107, 489–499. Available from: 10.1007/s00421-009-1136-0 19680682

[joa70025-bib-0005] Bergstrom, J. (1975) Percutaneous needle biopsy of skeletal muscle in physiological and clinical research. Scandinavian Journal of Clinical and Laboratory Investigation, 35, 609–616. 1108172

[joa70025-bib-0006] Blaauw, B. , Schiaffino, S. & Reggiani, C. (2013) Mechanisms modulating skeletal muscle phenotype. Comprehensive Physiology, 3, 1645–1687. Available from: 10.1002/cphy.c130009 24265241

[joa70025-bib-0007] Burke, S.K. , Fenton, A.I. , Konokhova, Y. & Hepple, R.T. (2021) Variation in muscle and neuromuscular junction morphology between atrophy‐resistant and atrophy‐prone muscles supports failed re‐innervation in aging muscle atrophy. Experimental Gerontology, 156, 111613. Available from: 10.1016/j.exger.2021.111613 34740815

[joa70025-bib-0008] Burkholder, T.J. , Fingado, B. , Baron, S. & Lieber, R.L. (1994) Relationship between muscle fiber types and sizes and muscle architectural properties in the mouse hindlimb. Journal of Morphology, 221, 177–190. Available from: 10.1002/jmor.1052210207 7932768

[joa70025-bib-0009] Condon, K. , Silberstein, L. , Blau, H.M. & Thompson, W.J. (1990) Development of muscle fiber types in the prenatal rat hindlimb. Developmental Biology, 138, 256–274. Available from: 10.1016/0012-1606(90)90196-p 2108065

[joa70025-bib-0010] Cotta, A. , Carvalho, E. , Da‐Cunha‐Júnior, A.L. , Valicek, J. , Navarro, M.M. , Junior, S.B. et al. (2021) Muscle biopsy essential diagnostic advice for pathologists. Surgical and Experimental Pathology, 4, 3. Available from: 10.1186/s42047-020-00085-w

[joa70025-bib-0011] Cotter, J.A. , Yu, A. , Kreitenberg, A. , Haddad, F.H. , Baker, M.J. , Fox, J.C. et al. (2013) Suction‐modified needle biopsy technique for the human soleus muscle. Aviation, Space, and Environmental Medicine, 84, 1066–1073. Available from: 10.3357/asem.3632.2013 24261060 PMC4050078

[joa70025-bib-0012] Deschrevel, J. , Andries, A. , Maes, K. , Peeters, J. , van Opstal, A. , Jiang, D. et al. (2024) Histological analysis of the gastrocnemius muscle in preschool and school age children with cerebral palsy compared with age‐matched typically developing children. American Journal of Physiology‐Cell Physiology, 326, C573–C588. Available from: 10.1152/ajpcell.00344.2023 38105751

[joa70025-bib-0013] Ebben, W.P. , Simenz, C. & Jensen, R.L. (2008) Evaluation of plyometric intensity using electromyography. Journal of Strength and Conditioning Research, 22, 861–868. Available from: 10.1519/JSC.0b013e31816a834b 18438229

[joa70025-bib-0014] Edström, L. & Nyström, B. (1969) Histochemical types and sizes of fibres in normal human muscles. A biopsy study. Acta Neurologica Scandinavica, 45, 257–269. Available from: 10.1111/j.1600-0404.1969.tb01238.x 4185305

[joa70025-bib-0015] Ferri, A. , Scaglioni, G. , Pousson, M. , Capodaglio, P. , Van Hoecke, J. & Narici, M.V. (2003) Strength and power changes of the human plantar flexors and knee extensors in response to resistance training in old age. Acta Physiologica Scandinavica, 177, 69–78. Available from: 10.1046/j.1365-201X.2003.01050.x 12492780

[joa70025-bib-0016] Fidziańska, A. (1980) Human ontogenesis. I. Ultrastructural characteristics of developing human muscle. Journal of Neuropathology and Experimental Neurology, 39, 476–486. 7217996

[joa70025-bib-0017] Gazendam, M.G.J. & Hof, A.L. (2007) Averaged EMG profiles in jogging and running at different speeds. Gait & Posture, 25, 604–614. Available from: 10.1016/j.gaitpost.2006.06.013 16887351

[joa70025-bib-0018] Gollnick, P.D. , Sjödin, B. , Karlsson, J. , Jansson, E. & Saltin, B. (1974) Human soleus muscle: a comparison of fiber composition and enzyme activities with other leg muscles. Pflugers Archiv: European Journal of Physiology, 348, 247–255. Available from: 10.1007/BF00587415 4275915

[joa70025-bib-0019] Häggmark, T. & Eriksson, E. (1979) Hypotrophy of the soleus muscle in man after achilles tendon rupture. Discussion of findings obtained by computed tomography and morphologic studies. The American Journal of Sports Medicine, 7, 121–126. Available from: 10.1177/036354657900700208 434290

[joa70025-bib-0020] Hamilton, M.T. , Hamilton, D.G. & Zderic, T.W. (2022) A potent physiological method to magnify and sustain soleus oxidative metabolism improves glucose and lipid regulation. iScience, 25, 104869. Available from: 10.1016/j.isci.2022.104869 36034224 PMC9404652

[joa70025-bib-0021] Hardy, E.J.O. , Inns, T.B. , Hatt, J. , Doleman, B. , Bass, J.J. , Atherton, P.J. et al. (2022) The time course of disuse muscle atrophy of the lower limb in health and disease. Journal of Cachexia, Sarcopenia and Muscle, 13, 2616–2629. Available from: 10.1002/jcsm.13067 36104842 PMC9745468

[joa70025-bib-0022] Hendrickse, P.W. , Wüst, R.C.I. , Ganse, B. , Giakoumaki, I. , Rittweger, J. , Bosutti, A. et al. (2022) Capillary rarefaction during bed rest is proportionally less than fibre atrophy and loss of oxidative capacity. Journal of Cachexia, Sarcopenia and Muscle, 13, 2712–2723. Available from: 10.1002/jcsm.13072 36102002 PMC9745458

[joa70025-bib-0023] Jensen, T.E. , Leutert, R. , Rasmussen, S.T. , Mouatt, J.R. , Christiansen, M.L.B. , Jensen, B.R. et al. (2012) EMG‐normalised kinase activation during exercise is higher in human gastrocnemius compared to soleus muscle. PLoS One, 7, e31054. Available from: 10.1371/journal.pone.0031054 22347426 PMC3275615

[joa70025-bib-0024] Joyce, N.C. , Oskarsson, B. & Jin, L.‐W. (2012) Muscle biopsy evaluation in neuromuscular disorders. Physical Medicine and Rehabilitation Clinics of North America, 23, 609–631. Available from: 10.1016/j.pmr.2012.06.006 22938878 PMC4590778

[joa70025-bib-0025] Kolk, S. , Klawer, E.M.E. , Schepers, J. , Weerdesteyn, V. , Visser, E.P. & Verdonschot, N. (2015) Muscle activity during walking measured using 3D MRI segmentations and [18F]‐Fluorodeoxyglucose in combination with positron emission tomography. Medicine and Science in Sports and Exercise, 47, 1896–1905. Available from: 10.1249/MSS.0000000000000607 25551402

[joa70025-bib-0026] Lieber, R.L. & Fridén, J. (2000) Functional and clinical significance of skeletal muscle architecture. Muscle & Nerve, 23, 1647–1666. Available from: 10.1002/1097-4598(200011)23:11<1647::aid-mus1>3.0.co;2-m 11054744

[joa70025-bib-0027] Luden, N. , Minchev, K. , Hayes, E. , Louis, E. , Trappe, T. & Trappe, S. (2008) Human vastus lateralis and soleus muscles display divergent cellular contractile properties. American Journal of Physiology. Regulatory, Integrative and Comparative Physiology, 295, R1593–R1598. Available from: 10.1152/ajpregu.90564.2008 18815206 PMC2584861

[joa70025-bib-0028] Mittendorfer, B. , Andersen, J.L. , Plomgaard, P. , Saltin, B. , Babraj, J.A. , Smith, K. et al. (2005) Protein synthesis rates in human muscles: neither anatomical location nor fibre‐type composition are major determinants. The Journal of Physiology, 563, 203–211. Available from: 10.1113/jphysiol.2004.077180 15611031 PMC1665563

[joa70025-bib-0029] Naruse, M. , Trappe, S. & Trappe, T.A. (2023) Human skeletal muscle‐specific atrophy with aging: a comprehensive review. Journal of Applied Physiology (Bethesda, Md.: 1985), 134, 900–914. Available from: 10.1152/japplphysiol.00768.2022 36825643 PMC10069966

[joa70025-bib-0030] Olesen, A.T. , Malchow‐Møller, L. , Bendixen, R.D. , Kjær, M. , Svensson, R.B. , Andersen, J.L. et al. (2021) Age‐related myofiber atrophy in old mice is reversed by ten weeks voluntary high‐resistance wheel running. Experimental Gerontology, 143, 111150. Available from: 10.1016/j.exger.2020.111150 33181317

[joa70025-bib-0031] Queeno, S.R. , Sterner, K.N. & O'Neill, M.C. (2023) Meta‐analysis data of skeletal muscle slow fiber content across mammalian species. Data in Brief, 50, 109520. Available from: 10.1016/j.dib.2023.109520 37701714 PMC10493253

[joa70025-bib-0032] Roberts, B.M. , Lavin, K.M. , Many, G.M. , Thalacker‐Mercer, A. , Merritt, E.K. , Bickel, C.S. et al. (2018) Human neuromuscular aging: sex differences revealed at the myocellular level. Experimental Gerontology, 106, 116–124. Available from: 10.1016/j.exger.2018.02.023 29481967 PMC6031257

[joa70025-bib-0033] Ross, L. , McKelvie, P. , Reardon, K. , Wong, H. , Wicks, I. & Day, J. (2023) Muscle biopsy practices in the evaluation of neuromuscular disease: a systematic literature review. Neuropathology and Applied Neurobiology, 49, e12888. Available from: 10.1111/nan.12888 36734037 PMC10946625

[joa70025-bib-0034] Saltin, B. , Henriksson, J. , Nygaard, E. , Andersen, P. & Jansson, E. (1977) Fiber types and metabolic potentials of skeletal muscles in sedentary man and endurance runners. Annals of the New York Academy of Sciences, 301, 3–29. Available from: 10.1111/j.1749-6632.1977.tb38182.x 73362

[joa70025-bib-0035] Schiaffino, S. & Reggiani, C. (1996) Molecular diversity of myofibrillar proteins: gene regulation and functional significance. Physiological Reviews, 76, 371–423. Available from: 10.1152/physrev.1996.76.2.371 8618961

[joa70025-bib-0036] Seynnes, O.R. , Maganaris, C.N. , de Boer, M.D. , di Prampero, P.E. & Narici, M.V. (2008) Early structural adaptations to unloading in the human calf muscles. Acta Physiologica (Oxford, England), 193, 265–274. Available from: 10.1111/j.1748-1716.2008.01842.x 18266998

[joa70025-bib-0037] Siebert, T. , Tomalka, A. , Stutzig, N. , Leichsenring, K. & Böl, M. (2017) Changes in three‐dimensional muscle structure of rabbit gastrocnemius, flexor digitorum longus, and tibialis anterior during growth. Journal of the Mechanical Behavior of Biomedical Materials, 74, 507–519. Available from: 10.1016/j.jmbbm.2017.07.045 28778781

[joa70025-bib-0038] Sjøgaard, G. (1982) Capillary supply and cross‐sectional area of slow and fast twitch muscle fibres in man. Histochemistry, 76, 547–555. Available from: 10.1007/BF00489909 7166513

[joa70025-bib-0039] Soendenbroe, C. , Dahl, C.L. , Meulengracht, C. , Tamáš, M. , Svensson, R.B. , Schjerling, P. et al. (2022a) Preserved stem cell content and innervation profile of elderly human skeletal muscle with lifelong recreational exercise. The Journal of Physiology, 600, 1969–1989. Available from: 10.1113/JP282677 35229299 PMC9315046

[joa70025-bib-0040] Soendenbroe, C. , Heisterberg, M.F. , Schjerling, P. , Karlsen, A. , Kjaer, M. , Andersen, J.L. et al. (2019) Molecular indicators of denervation in aging human skeletal muscle. Muscle & Nerve, 60, 453–463. Available from: 10.1002/mus.26638 31314910

[joa70025-bib-0041] Soendenbroe, C. , Heisterberg, M.F. , Schjerling, P. , Kjaer, M. , Andersen, J.L. & Mackey, A.L. (2022b) Human skeletal muscle acetylcholine receptor gene expression in elderly males performing heavy resistance exercise. American Journal of Physiology. Cell Physiology, 323, C159–C169. Available from: 10.1152/ajpcell.00365.2021 35649253

[joa70025-bib-0042] Soendenbroe, C. , Karlsen, A. , Svensson, R.B. , Kjaer, M. , Andersen, J.L. & Mackey, A.L. (2024) Marked irregular myofiber shape is a hallmark of human skeletal muscle ageing and is reversed by heavy resistance training. Journal of Cachexia, Sarcopenia and Muscle, 15, 306–318. Available from: 10.1002/jcsm.13405 38123165 PMC10834339

[joa70025-bib-0043] Trappe, S. , Creer, A. , Minchev, K. , Slivka, D. , Louis, E. , Luden, N. et al. (2008) Human soleus single muscle fiber function with exercise or nutrition countermeasures during 60 days of bed rest. American Journal of Physiology. Regulatory, Integrative and Comparative Physiology, 294, R939–R947. Available from: 10.1152/ajpregu.00761.2007 18094071

[joa70025-bib-0044] Trappe, T.A. , Raue, U. & Tesch, P.A. (2004) Human soleus muscle protein synthesis following resistance exercise. Acta Physiologica Scandinavica, 182, 189–196. Available from: 10.1111/j.1365-201X.2004.01348.x 15450115

[joa70025-bib-0045] Viguie, C.A. , Lu, D.‐X. , Huang, S.‐K. , Rengen, H. & Carlson, B.M. (1997) Quantitative study of the effects of long‐term denervation on the extensor digitorum longus muscle of the rat. The Anatomical Record, 248, 346–354. Available from: 10.1002/(SICI)1097-0185(199707)248:3<346::AID-AR7>3.0.CO;2-N 9214552

[joa70025-bib-0046] Ward, S.R. , Eng, C.M. , Smallwood, L.H. & Lieber, R.L. (2009) Are current measurements of lower extremity muscle architecture accurate? Clinical Orthopaedics and Related Research, 467, 1074–1082. Available from: 10.1007/s11999-008-0594-8 18972175 PMC2650051

[joa70025-bib-0047] Wick, C. , Böl, M. , Müller, F. , Blickhan, R. & Siebert, T. (2018) Packing of muscles in the rabbit shank influences three‐dimensional architecture of *M. soleus* . Journal of the Mechanical Behavior of Biomedical Materials, 83, 20–27. Available from: 10.1016/j.jmbbm.2018.04.006 29656240

[joa70025-bib-0048] Willingham, T.B. , Kim, Y. , Lindberg, E. , Bleck, C.K.E. & Glancy, B. (2020) The unified myofibrillar matrix for force generation in muscle. Nature Communications, 11, 3722. Available from: 10.1038/s41467-020-17579-6 PMC7381600 32709902

[joa70025-bib-0049] Winter, D.A. & Yack, H.J. (1987) EMG profiles during normal human walking: stride‐to‐stride and inter‐subject variability. Electroencephalography and Clinical Neurophysiology, 67, 402–411. Available from: 10.1016/0013-4694(87)90003-4 2444408

[joa70025-bib-0050] Yamazaki, Y. , Suzuki, M. & Mano, T. (1993) Control of rapid elbow extension movement. Brain Research Bulletin, 30, 11–19. Available from: 10.1016/0361-9230(93)90034-9 8420620

