# Supplementary figures and images for: Morphological differences in myofibre size and shape: A comparative study of the soleus, gastrocnemius, triceps brachii and vastus lateralis in humans and mice
Source: J Anat. 2025 Jul 17;248(1):126–39. doi: 10.1111/joa.70025 (PMC12682596; doi:10.1111/joa.70025)

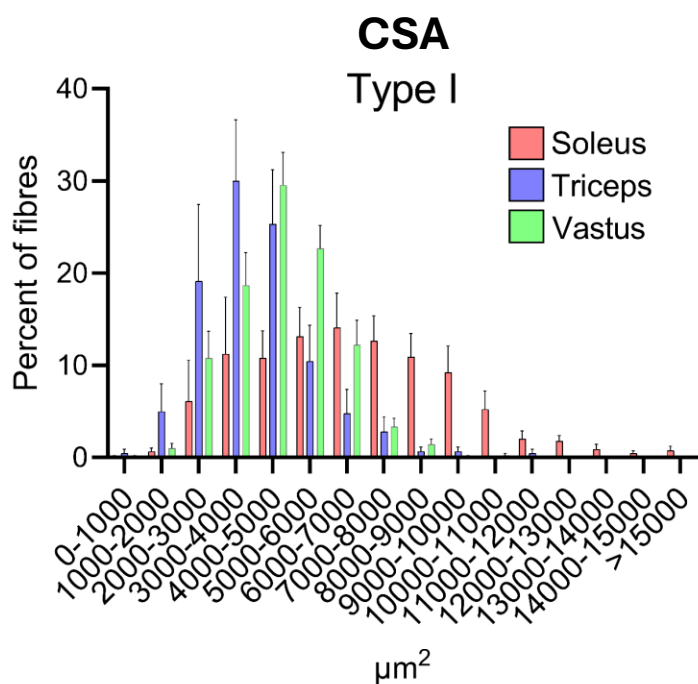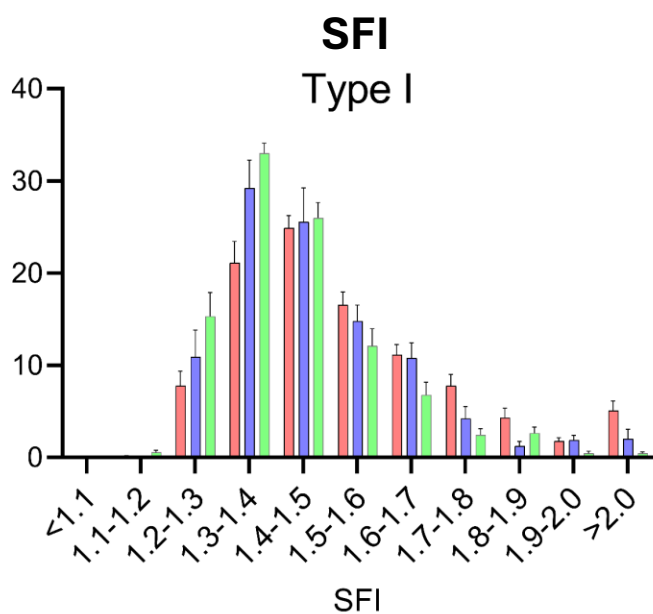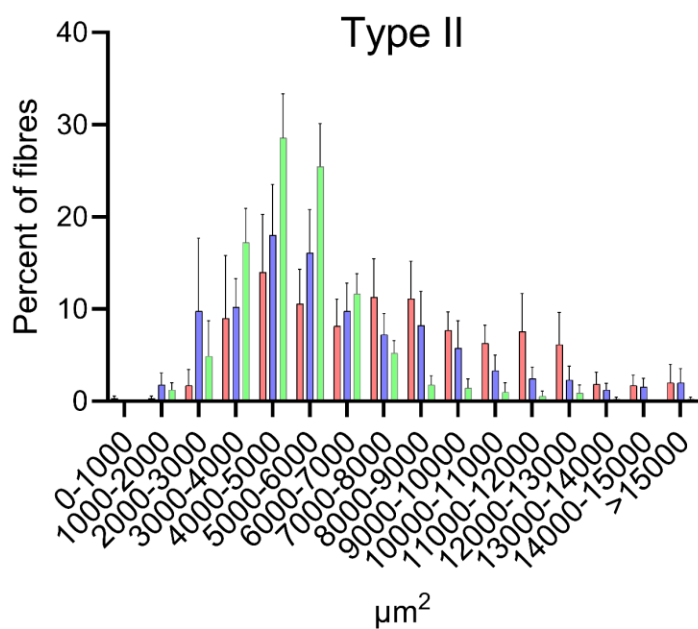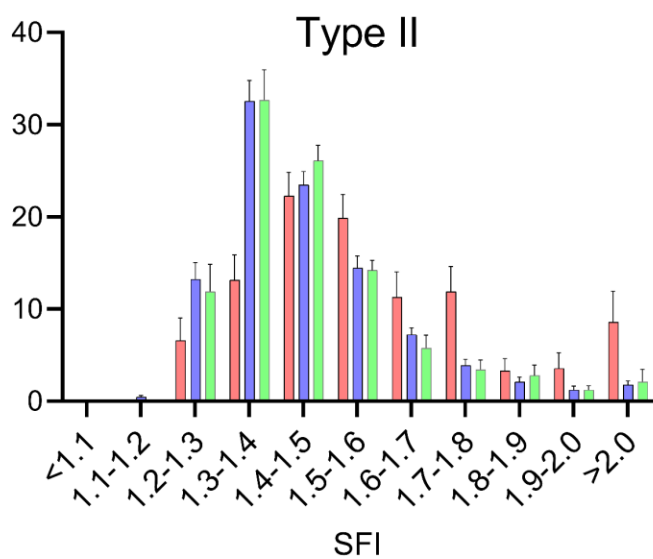

Supplement: Supplementary file 1 — Figure S1. CSA and SFI distribution of type I and II fibres of soleus (red), triceps brachii (blue) and vastus lateralis (green) of 9 young healthy men. (A‐B) Percentage of type I (A) and II (B) myofibres in 1000 μm2 increments of CSA. (C‐D) Percentage of type I (C) and II (D) myofibres in 0.1 increments of SFI. Data are averages of all participants for each muscle and presented as means ± SEM. N: Type I = 9 in all muscles. Type II = 7 in Sol, 9 in Tri and Vas. Abbreviations: CSA, cross‐section area. SFI, shape factor index. [file JOA-248-126-s001.pdf]
